# PyViscount: Validating False Discovery Rate Estimation Methods via Random Search Space Partition

**DOI:** 10.1021/acs.jproteome.4c00743

**Published:** 2025-02-05

**Authors:** Dominik Madej, Henry Lam

**Affiliations:** Department of Chemical and Biological Engineering, The Hong Kong University of Science and Technology, Hong Kong 999077, China

**Keywords:** validation, false discovery rate, search space partition, peptide identification, shotgun proteomics

## Abstract

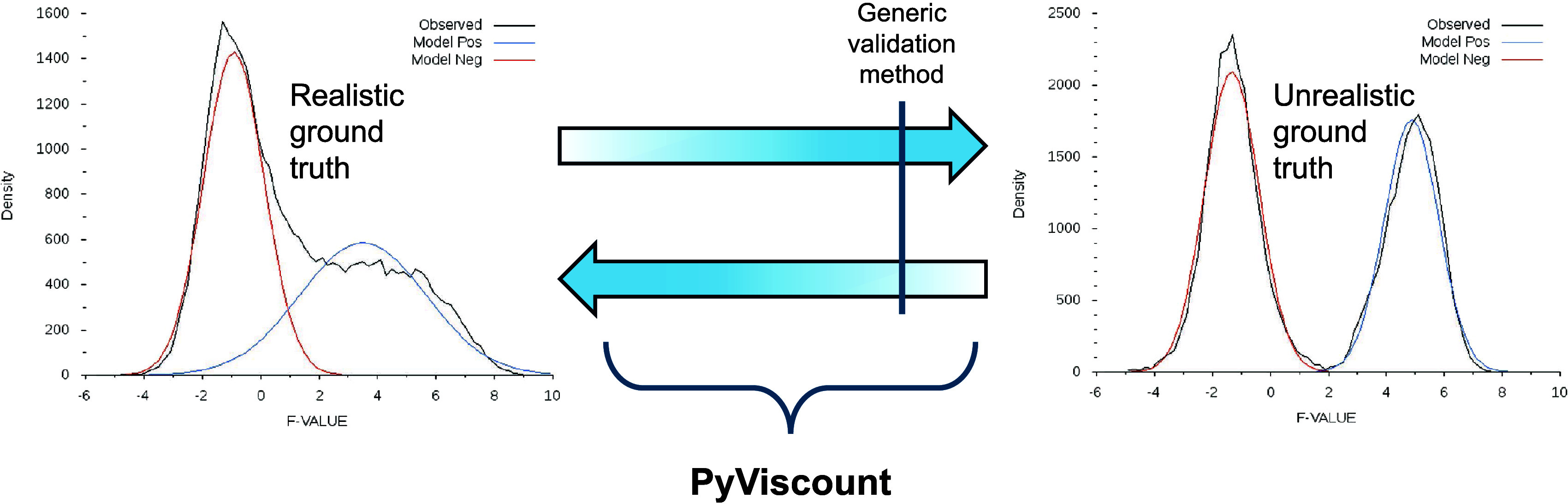

Validating false discovery rate (FDR) estimation is an essential but surprisingly understudied aspect of method development in shotgun proteomics. Currently available validation protocols mostly rely on ground truth data sets, which typically involve manipulating the properties of the search space or query spectra used. As a result, comparing estimated FDR and ground truth-based false discovery proportion values may not be representative of the scenarios involving natural data sets encountered in practice. In this study, we introduce PyViscount—a Python tool implementing a novel validation protocol based on random search space partition, which enables generating a quasi ground-truth using unaltered search spaces of unique candidate peptides and generic data sets of experimental query spectra. Furthermore, validation of existing FDR estimation methods by PyViscount is consistent with alternative validation protocols. The presented novel approach to validation free from the need for synthetic data sets or dubious manipulation of the data may be an attractive alternative for proteomics practitioners, allowing them to obtain deeper insights into the performance of existing and new FDR estimation methods.

## Introduction

The validation of a false discovery rate (FDR)^1,2^ estimation method aims to verify the accuracy of the provided estimates by comparing them with gold standard or high-confidence reference metric values under specific conditions. It is particularly important in shotgun proteomics, which typically deals with complex experimental data that undergoes multiple preprocessing steps before being subjected to one of many available FDR estimation methods.^3^ Analyses with such characteristics may not always meet the theoretical assumptions or other requirements of the FDR estimation procedure selected.^4−6^ Hence, it is crucial to investigate whether potential violations of these requirements significantly impact the FDR estimates, especially in the case of “black-box” machine learning approaches.^7^ Finally, some aspects of the data analysis workflow used may be unknown to the researcher but still influence the accuracy and robustness of the FDR estimation. Then, the only way to identify potential issues with such procedures is to conduct validation and explicitly evaluate the estimated and expected metric values.

Validation of statistical confidence of spectral matches in shotgun proteomics can be approached from different angles. Some methods focus on comparing the estimated FDR and false discovery proportion (FDP) values directly, while others assess the quality of metrics required for FDR estimation. Determination of FDP virtually always relies on search results obtained for a ground truth data set, i.e., a collection of spectra that give rise to unambiguously incorrect (negative samples) or correct matches (positive samples).

The most popular validation protocols utilize information about both correct and incorrect identifications. One of the earliest, although not designed for estimating FDR per se, was manual validation.^8^ Researchers had to look at each peptide-spectrum match and, based on their expertise, decide if it was correct or incorrect. This time-consuming and subjective approach was quickly recognized as suboptimal, and the search for more objective, standardized methods continued.

Once the field steered away from manual validation and simple heuristics-based solutions such as applying arbitrary cutoff score thresholds, the need for proper ground truth data sets suitable for assessing statistical error control procedures appeared. Over the years, several methods of preparing ground truth data sets have been developed. Generation of positive samples (giving rise to correct identifications) is a relatively straightforward task and most frequently achieved by searching the selected spectral data set with multiple search engines against the same search space and identifying the spectra whose top-scoring candidates do not differ among the evaluated search variants.^9,10^ Alternatively, the ground truth data set may involve spectra of synthetic peptides^11,12^ or natural peptides from a purified protein mix.^13−15^ In those cases, spectra that produce matches to sequences of peptides known to be present in the analyzed biological mixture are considered positive samples. This approach is central to validation procedures utilizing entrapment databases.^6,16−18^ Positive samples can also be taken directly from high-quality spectral libraries (consensus spectra) or generated in silico by various mass spectra simulators.^19−22^

Preparing negative samples for the ground truth data sets is more challenging because it is difficult to establish what constitutes an unambiguous and realistic incorrect spectral match. Many procedures proposed to achieve that rely on manipulating features of the spectral queries or the search space. For instance, the protocol outlined in the pValid study^9^ for closed search settings involves shifting the precursor mass of query spectra. This adjustment ensures that the selected pool of peptide candidates used in scoring those queries does not contain any correct candidates. Another approach utilizes shuffling all sequences in the search space except those corresponding to correct matches of the already prepared set of positive samples.^10^ Any spectra matched to these shuffled sequences are then considered negative samples. The frameworks utilizing entrapment databases rely on a similar idea—a large set of peptide candidates from a foreign organism (or shuffled sequences of the original organism) are appended to the original sample search space and are assumed to trap virtually all incorrect spectral matches by outcompeting any sample candidates.^16^

Generating negative samples also plays a central role in validation protocols that focus not on FDP calculation but on assessing the quality of the metrics used for FDR estimation. While these approaches are less common and do not verify the accuracy of FDR estimates directly, they can still provide meaningful information on how well an FDR estimation framework satisfies certain requirements. The most common form of such validation involves verifying the uniformity of *p*-values of spectral matches under the null hypothesis. In practice, it can be achieved by comparing *p*-values of interest against *p*-values obtained from the semilabeled analysis of entrapment database search results^6^ or from decoy peptide-spectrum match (PSMs).^23^ Quality assessment of *p*-values may provide some insights into the performance of the FDR estimation methods that rely on those *p*-values, e.g., the Benjamini–Hochberg procedure^1^ and its many variants.

While all approaches using modification of queries or search space to enable the construction of ground truth data sets have their specific problems and limitations, they have one issue in common. To some extent, they render the ground truth data set unrealistic by introducing artificial changes to the query spectra or peptide candidates. That, in turn, invites the question of how reliable are the validation results based on such ground truth data sets, since there is no guarantee they will accurately represent the FDR estimation trends in analyses involving natural experimental spectra and unaltered search spaces. Similar concerns could be raised for data sets of synthetic origins or controlled composition.

From a wider perspective, the problem of unrealistic validation arises from using factors directly involved in both generating the evaluated spectral matches to construct the ground truth and determining the exact identification status labels (correct/incorrect match) of those matches. Therefore, it would be desirable to have a validation protocol in which factors generating the exact identification status labels do not interfere with the natural characteristics of the analyzed data. While this objective is rarely stated explicitly, its traces can be found in some studies. For example, in the validation context, Dowell and colleagues used the fold change difference in a quantification study of a two-organism protein mixture to verify the identity of reported peptides and proteins.^24^ The theme of using factors that do not interfere with the identity of PSMs also appears in the study on “FDR-like functions” employing PSM features orthogonal to the conventional similarity scores.^25^

**Figure 1:**
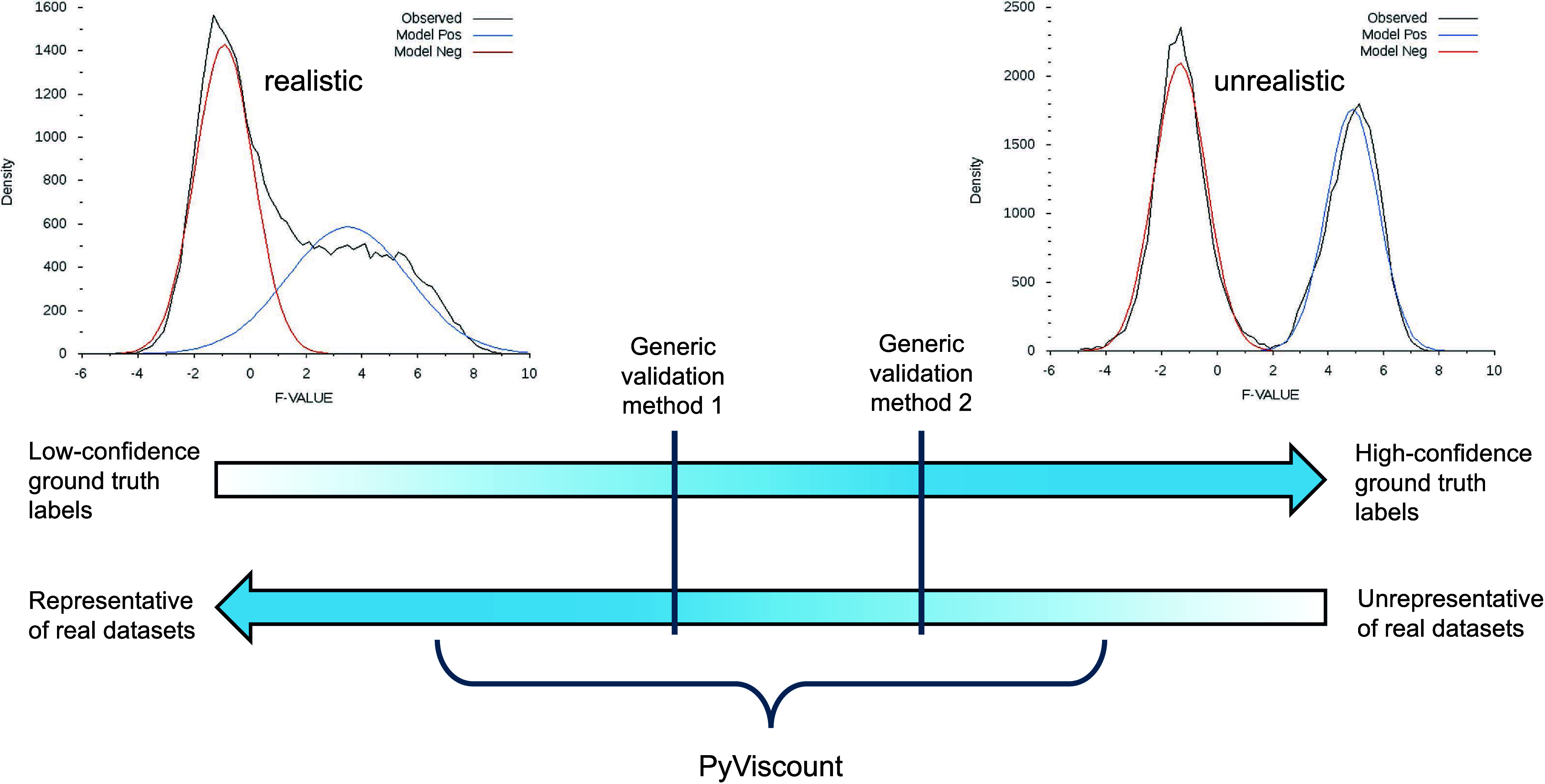
Characteristics of ground truth data sets used by validation frameworks in shotgun proteomics.

Validation frameworks based on the exact identification status labels can be imagined as points on the continuum (Figure 1). One end of that continuum represents the situation in which the validation relies on a ground truth data set with a high degree of artificiality typical for synthetic, simulated, or heavily curated data sets. The nature of these data sets enables pinpointing the identification status labels with high confidence. However, their practical utility may be limited since they may not represent natural data sets well. The other end of the continuum represents the opposite scenario involving an unaltered and thus realistic data set, which comes at the cost of uncertainty regarding the identity of the matches produced for that data set. Therefore, validation methods located on that end of the spectrum cannot provide reliable results either. Existing validation protocols in proteomics are usually designed to work under specific, fixed conditions and thus represent only several possible validation scenarios along the discussed continuum. Even when a validation protocol is well-described, it may not be straightforward to pinpoint its location on the continuum and compare it against other validation alternatives.

Considering the characteristics of methods discussed in the preceding paragraphs of this introduction, it is clear that shotgun proteomics has a limited arsenal of tools to validate FDR estimation frameworks flexibly and comprehensively. The varying degrees of artificiality, narrow selection of compatible data sets, and small range of the conditions covered prevent the existing solutions from delivering optimal validation results. Validation frameworks free from those problems would be highly beneficial to the community.

This article presents a novel protocol for validating FDR estimation methods in shotgun proteomics. The proposed method relies on a random search space partition to enable determining the identification status labels crucial for calculating FDP used to evaluate the accuracy of FDR estimates. The random partition does not interfere with the original characteristics of the search space, is compatible with generic spectral data sets, and makes it possible to investigate various search scenarios. The presented validation protocol is the basis of a newly developed command-line Python tool called PyViscount, which enables validation of major FDR estimation frameworks executed on user-provided search results from popular search engines or postprocessors.

## Methods

### Validation by Search Space Partition

The proposed validation protocol is built on three fundamental premises that enable determining identification status labels (correct/incorrect) of spectral matches necessary for calculating false discovery proportion.

The first premise is that it is possible to reduce the fraction of unidentifiable spectra (e.g., those lacking correct peptide candidates in the original search space considered) in the query spectra set by discarding spectra whose top-scoring candidates fail to pass a selected similarity score threshold. This quality filtering (QF) step retains a set of spectra whose top-scoring candidates from the full search space are most likely correct and thus can be treated as positive samples in the ground truth construction. Furthermore, it can be applied across a range of threshold values, so that when the threshold gets higher, the fraction of identifiable spectra in the filtered set increases. Consequently, we can generate positive sample sets associated with progressively increasing confidence of the corresponding identification status labels. The user can specify the range of QF threshold values, which must ensure that the number of spectra retained after filtering with the upper bound of the range produces a large enough set of PSMs in the search executed on the subset of the original search space (from now on referred to as “subset search”). This condition is necessary to produce sufficiently smooth pFDP vs FDR lines in the final plot, and we recommend the users keep at least a thousand PSMs in the subset search.

The second notion is that the trends observed when searching a subset of a randomly partitioned search space are consistent with those obtained from searching the whole original search space. This point is necessary to make since the proposed validation framework relies on searching a subset of the partitioned search space to calculate FDP. It is worth mentioning that the framework is compatible with any search space that can be represented as a set of unique peptide candidates, e.g., a protein/peptide sequence database, a spectral library, or potentially even a spectral archive.

The third premise is that searching the spectra remaining after the quality filtering (QF) step against a subset of the search space partition generates some unambiguously incorrect matches, which may constitute negative samples for the ground truth. Some spectra retained after the quality filtering step will inevitably have their top-scoring candidates (obtained from the whole original search space) absent from the selected subset of the search space partition. As a result, any candidates matched to these spectra in the subset search are presumed to be unambiguously incorrect, assuming that each spectrum can have only one correct peptide candidate and if it exists in the search space, it always becomes the top hit. For the remaining spectra, whose top-scoring candidates are still present in the search space subset, the top matches remain the same as in a whole search space search and can be considered putatively correct (positive samples).

While we can confidently determine the identification status of incorrect matches produced in the partition subset search, the status of the correct matches is more uncertain because it depends on the effectiveness of the QF step. Setting the quality threshold too low may result in retaining many unidentifiable spectra and marking them as identifiable (having correct candidates in the search space). Conversely, a threshold set too high will limit the number of spectra available for the partition subset search and lead to highly variable results with little utility. Hence, it seems more reasonable to evaluate multiple validation scenarios based on data obtained from filtering conducted on a range of quality score threshold values.

The proposed validation protocol may involve partitioning the search space first (presearch mode) or partitioning the results of the whole search space search only (postsearch mode).

### Presearch Partition Mode

In the first step of the presearch partition mode, the experimental spectra in the query set *Q* are matched against the full set of peptide candidates Ω. This creates a list of matches between spectra and peptides. Only spectra with a top match that exceeds a user-specified similarity score threshold are kept. This filtering step produces a smaller, higher-quality subset of spectra, *Q_f_*.

Then, the full set of peptides Ω is partitioned into multiple equal-sized disjoint subsets based on a number chosen by the user, and one of these subsets is randomly picked for further analysis. The filtered spectra (*Q_f_*) are then matched against the selected subset of peptides. A new list of matches is created for these comparisons. The top-matching peptide for each spectrum is identified using the same scoring method, and the results are compared to the original matches from the full set. If the top match from the subset search agrees with the original top match, the peptide-spectrum match is marked as correct. Otherwise, it is considered incorrect.

**Figure 2:**
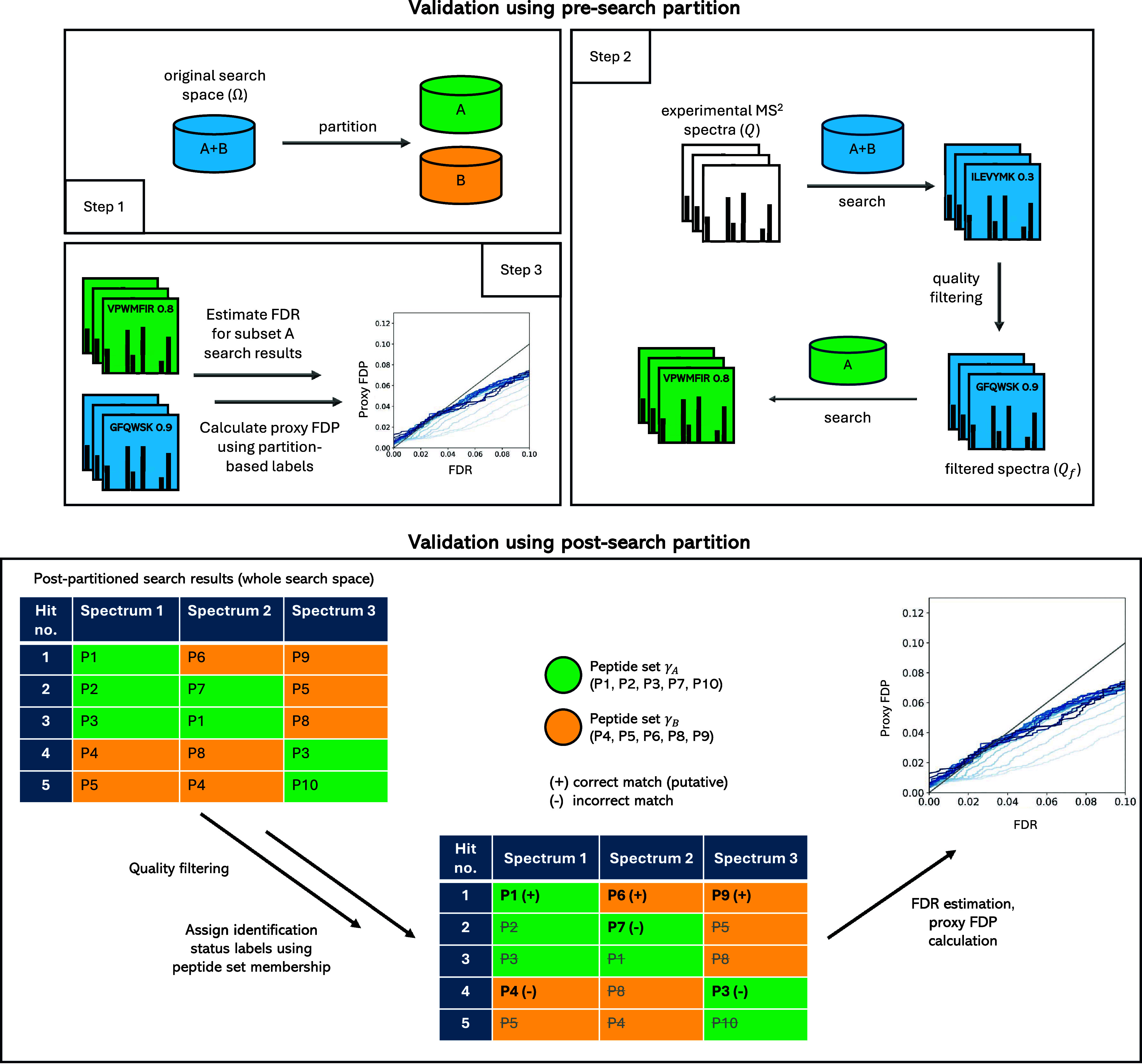
Workflows of validation using PyViscount in presearch and postsearch partition modes. In the presearch mode, experimental spectra (*Q*) are matched against all peptide candidates (Ω) and filtered by a user-selected similarity score threshold to form *Q*_f_. These filtered spectra (*Q_f_*) are then matched against a randomly selected subset of Ω. The resulting peptide-spectrum matches (PSMs) are compared with the original matches to determine identification status (correct/incorrect) and calculate the proxy false discovery proportion (pFDP). In the postsearch mode, spectra are matched against Ω and filtered by a similarity score threshold. The top 10 peptide candidates for each filtered spectrum are partitioned into sets γ_A_ and γ_B_. By considering the highest-scoring matches in the selected γ set (and ignoring the rest, marked as dashed out gray in the table), identification status labels are established. Matches that are top hits in the Ω search are marked as putatively correct, while others are considered incorrect. The proxy FDP is then calculated and compared against FDR. The same process in either post- or presearch mode can be repeated for different values of user-specified score threshold in the quality filtering step to produce “ground truth” PSMs of varying quality.

The PSMs produced in this process are then used to calculate a metric called the proxy false discovery proportion (pFDP), representing the proportion of incorrect matches among the top-scoring matches that exceed a set validation score threshold. Finally, the calculated pFDP is compared with the corresponding FDR estimates, and the whole process is repeated for multiple *Q_f_* sets obtained for a range of similarity score thresholds. A simplified schematic of the described validation protocol in the presearch partition mode is presented in Figure 2.

It is worth clarifying that pFDR is used instead of FDP because the confidence of identification status labels of matches marked as correct depends on how stringent the quality filtering step is. Once the similarity score threshold is high enough to retain only high-quality spectra in the *Q_f_* set, the values of pFDP and FDP will be virtually identical.

### Postsearch Partition Mode

The first steps of the postsearch validation are similar to those in the presearch approach. First, the query spectra set *Q* is searched against the entire set of possible peptide candidates (Ω), resulting in a list of matches between spectra and peptides. A quality filtering step is applied to retain only high-confidence spectra, producing a filtered subset (*Q_f_*) for further analysis.

At this point, the process diverges from the presearch approach. Instead of dividing the entire peptide search space (Ω) into subsets, the partitioning is performed on the list of top matches obtained for each spectrum in *Q_f_*. Specifically, the set of unique peptide candidates is created from the top *N* matches for each spectrum, where *N* is chosen by the user (typically *N* ≥ 10 to ensure sufficient size). This set is then divided into equal-sized subsets. Only one of these subsets (γ_A_) is selected for the next steps of the analysis.

In this “simulated” search, the highest-scoring match from γ_A_ is determined for each spectrum in *Q_f_* by scanning through the list of top *N* candidates. If that match is the same as the best match from the original full search, the spectrum is labeled as correctly identified. If the peptide is found in γ_A_ but is not the best candidate in the full search, it is labeled as incorrect. In rare cases, if none of the top *N* candidates for a spectrum belongs to γ_A_ (due to the random nature of the partitioning process), that spectrum is excluded from further analysis.

Once all spectra in *Q*_f_ have been labeled as correct or incorrect, the pFDP is calculated and compared with FDR estimates obtained using the evaluated method on the “simulated” search results of γ_A_ set. The simplified workflow of the validation based on the postsearch partition is presented in Figure 2.

### Verifying the Merits of Quality Filtering

Another assumption required for validation by partition is that by applying a progressively stringent quality filtering step, an increasing fraction of unidentifiable spectra can be removed from the spectral data set, up to the point when only spectra producing correct matches with high-confidence identification status are retained. This assumption can be verified by the following procedure utilizing the human protein sequence database (UniProt ID: UP000005640) and a data set of synthetic human spectra taken from the ProteomeTools project.^11^

First, the protein sequence database is randomly partitioned into two equal-sized sets, and one of those subsets (γ_A_) is selected for further analysis. Then, synthetic human spectra are searched against the full protein sequence database using Comet search engine,^26^ and the top-scoring PSMs are recorded. The next step is quality filtering, i.e., removing spectra whose top-scoring matches do not pass the user-defined quality threshold. The filtered spectra are then subjected to the subset search against γ_A_, and the top-scoring PSMs are recorded. The matches whose peptide candidate is the same in the γ_A_ search and the full protein sequence database are marked as correct, and the remaining ones as incorrect. In the last step of the procedure, the score distribution of matches marked as incorrect is compared with the distribution of matches whose top-scoring candidates do not belong to the list of synthetic peptides used to generate the ProteomeTools data set. The comparison is executed in the form of Kolmogorov–Smirnov (KS) test to check whether the empirical null distribution proposed by the validation protocol is significantly different from the actual null distribution of synthetic incorrect matches; the corresponding statistic and *p*-values are recorded for scenarios with different score thresholds applied in the quality filtering step.

### Validation of Decoy-Based FDR Estimation Methods

In this part of the study, the utility of PyViscount was demonstrated by validating two decoy-based FDR estimation methods: target-decoy competition (TDC) and Storey’s method^27^ using decoy-based *p*-values obtained from separate target-decoy search results (STD-P). The generic data set employed in this analysis was the Chinese hamster data set acquired on Q Exactive HF (PXD008760, file name: FE_CHO_D1_50 min5–45_4of15_20160205_r3.raw) searched against the corresponding target protein sequence database (UniProt ID: UP000001075) using Tide and MSFragger search engines, and against a Chinese hamster spectral library (NIST, peptidew:lib:cho_20180223) using SpectraST.^28^ All searches were executed with default search settings.

TDC involved searching the spectral data set against a concatenated target-decoy search space. In the case of Tide, the corresponding shuffled decoy sequences were generated using the tide-index tool from Crux suite^29^ with default settings; the concatenated target-decoy search was executed using tide-search command with the “concat” option set to “true”. When MSFragger was used, the concatenated target-decoy database was generated using decoyFastaGenerator.pl script provided in TPP suite.^30^ In the spectral library searching variant, the target-decoy spectral library was constructed by SpectraST according to the shuffle-reposition method^31^ using the NIST Chinese hamster spectral library. Whenever a search space-dependent score was used for FDR estimation (e.g., the transformed *e*-value score, TEV), the score value was adjusted to account for the decreased search space size in the subset search (details of the procedure are outlined in Supporting Information Note S1). For each search engine, the TDC-based FDR estimate  was calculated according to the formula ensuring unbiased estimation^32^
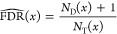
1where *N*_D_(*x*) and *N*_T_(*x*) are the numbers of decoy and target spectral matches, respectively, with similarity scores larger or equal to the selected threshold *x*.

The STD-P framework was evaluated by using separate search results against target and decoy search spaces for each studied search engine. The decoy variants of the protein sequence database and the spectral library were generated as outlined in the previous paragraph. When STD-P framework was used to estimate FDR, the top-scoring decoy matches were first collected, and the corresponding decoy-based *p*-values for target matches were calculated according to the formula recommended by Granholm and Käll^33^
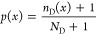
2

where *x* is the score of the evaluated spectral match, *p*(*x*) is the corresponding decoy-based p-value, *n*_D_(*x*) is the number of decoy matches with scores larger or equal to *x*, and *N*_D_ is the total number of decoy matches considered. These empirical *p*-values were then used as an input to Storey’s framework^27^ for FDR estimation.

In all search cases, the search engines were set to output the top 10 hits per spectrum to enable the postsearch validation by partition. Results of validating all investigated FDR estimation methods by PyViscount were presented in the form of a proxy FDP vs FDR contour plot (an example is shown in Supporting Information Figure S1). In that plot, each line represents proxy FDP vs FDR data obtained for a validation scenario with a particular value of QF score threshold taken from a predetermined wide range of values that can cover scenarios of validation with low- and high-quality identification status labels (details are described in Supporting Information Note S2). That way of reporting is particularly insightful at this stage of analysis as it illustrates how the validation trends change as the quality of the “ground truth” labels increases. Separate plots were generated for FDR generated on the PSM level and peptide level (only top-scoring PSM for each peptide considered).

### Validation of Decoy-Free FDR Estimation Methods

To further demonstrate its utility, PyViscount was used to validate three decoy-free FDR estimation variants on Tide search results executed using the Chinese hamster data set (the same as in the previous section) searched against the respective target protein sequence database. All evaluated scenarios utilized Storey’s protocol to estimate FDR but differed in the source of the *p*-values used. The first protocol (“Sidak-P”) employed the spectrum-level exact *p*-values calculated using the dynamic programming protocol implemented in Tide as described in.^34^ The *p*-values produced in that way were then adjusted using Šidák correction.^35^ The second protocol (“CDD-P”) derived *p*-values for the target matches by using Common Decoy Distributions (CDDs) as already constructed and published in the previous study.^36^ The transformed *e*-value (TEV) score that forms the basis of the CDDs was calculated according to the formula
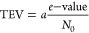
3where *a* = −0.02 and *N*_0_ = 1000.

The last protocol (“Lower-P”) used *p*-values generated from the TEV models of top-scoring incorrect target PSMs constructed using the information provided by the lower-scoring spectral matches.^37^

Analogously to the analysis of decoy-based FDR estimation methods, Tide was set to output the top 10 hits per spectrum to enable the postsearch validation by partition of the selected decoy-free protocols. All validation results were presented in the form of pFDP vs FDR contour plot, with separate plots generated for PSM- and peptide-level FDR estimates. In all evaluated cases, PyViscount used TEV as the QF score and estimated FDR according to Storey’s procedure.

### Comparison with Other Validation Protocols

The validation by partition was compared with three alternatives based on (i) synthetic peptide data set, (ii) entrapment database search, and (iii) intersection of search results from multiple search engines coupled with shifting precursor mass of some query spectra. In each validation scenario, the analyzed spectral data set was searched against a concatenated target-decoy search space, with the decoy component generated from the target counterpart using the tide-index tool from the Crux suite with default settings. All validation variants were used to compute FDP values. These values were then compared with the respective target-decoy competition (TDC)-based^38^ FDR estimates on both PSM and peptide levels. Finally, analogous validation of the same FDR estimation results was executed using PyViscount in a postsearch partition mode. To facilitate a fair comparison of validation provided by PyViscount and other methods, the PyViscount results were presented in the optimized single-line plot, where the most stable scenario out of many tested (as reported in the contour plot) was plotted along with the bootstrapped 68% pointwise confidence bands. This reporting form is also more useful when the user needs a simple plot for further analyses rather than a more diagnostic contour plot. The details of the procedure for constructing an optimized single-line plot are presented in Supporting Information Note S3 and Figures S2 and S3.

#### Entrapment Databases

The validation using entrapment databases was executed according to the popular protocol used in past studies.^16,18^ The ProteomeTools data set (same as in the previous paragraph) was searched against a bipartite database of sample sequences corresponding to the synthetic peptides from the data set and an entrapment component comprising a large number of protein sequences from a foreign organism, in this case *Arabidopsis thaliana* (39,281 protein sequences). The search was conducted using Tide with default settings. The rationale behind this approach is that the entrapment organism and the sample organism proteomes have negligible overlap. Since the entrapment set is typically selected to be much larger than the sample counterpart, any incorrect match is likely to be “trapped” by sequences originating from the foreign organism. In this context, matches mapped to entrapment sequences were marked as incorrect, while matches to sample sequences were deemed correct. Identification status labels of these spectral matches were subsequently used to calculate FDP.

#### Intersection of Results from Multiple Search Engines + Precursor Mass Shift

The validation using the intersection of multiple engine search results and precursor mass shift of spectra was conducted following the approach presented in the pValid study.^9^ First, the Chinese hamster data set used in the previous sections (PXD008760) was searched against the corresponding Chinese hamster target protein sequence database (UniProt ID: UP000001075) using three different search engines: Tide,^39^ MSFragger,^40^ and Comet,^41^ all with default settings. Then, the top-scoring matches produced for each spectrum were compared. A PSM was considered correct if its spectrum was assigned with the same top-scoring candidate by all three search engines. PSMs that did not fulfill this criterion had their precursor mass shifted by a fixed number (19.5 Th) and were researched. Any matches produced by these mass-shifted spectra were considered incorrect. Finally, the identification status labels of the correct and incorrect spectral matches generated according to the described protocol were used to calculate FDP.

#### Verification of Synthetic Origin

The validation based on verification of synthetic origin involved searching a randomly selected file with spectra of synthetic peptides produced in the ProteomeTools project (PXD004732, file name: 01625b_GB6-TUM_first_pool_42_01_01–3xHCD-1h-R1.raw) against a human target protein sequence database (UniProt ID: UP000005640) including the sample sequences of peptides used in the synthesis. This search was executed using Tide with default settings. To determine the identification status labels required for calculating FDP, we assessed whether the top-scoring matches were mapped to sequences from the list of synthetic peptides. Matches associated with such sequences were marked as correct, while the remaining matches were considered incorrect.

## Results and Discussion

### Merits of the Quality Filtering Step

**Figure 3:**
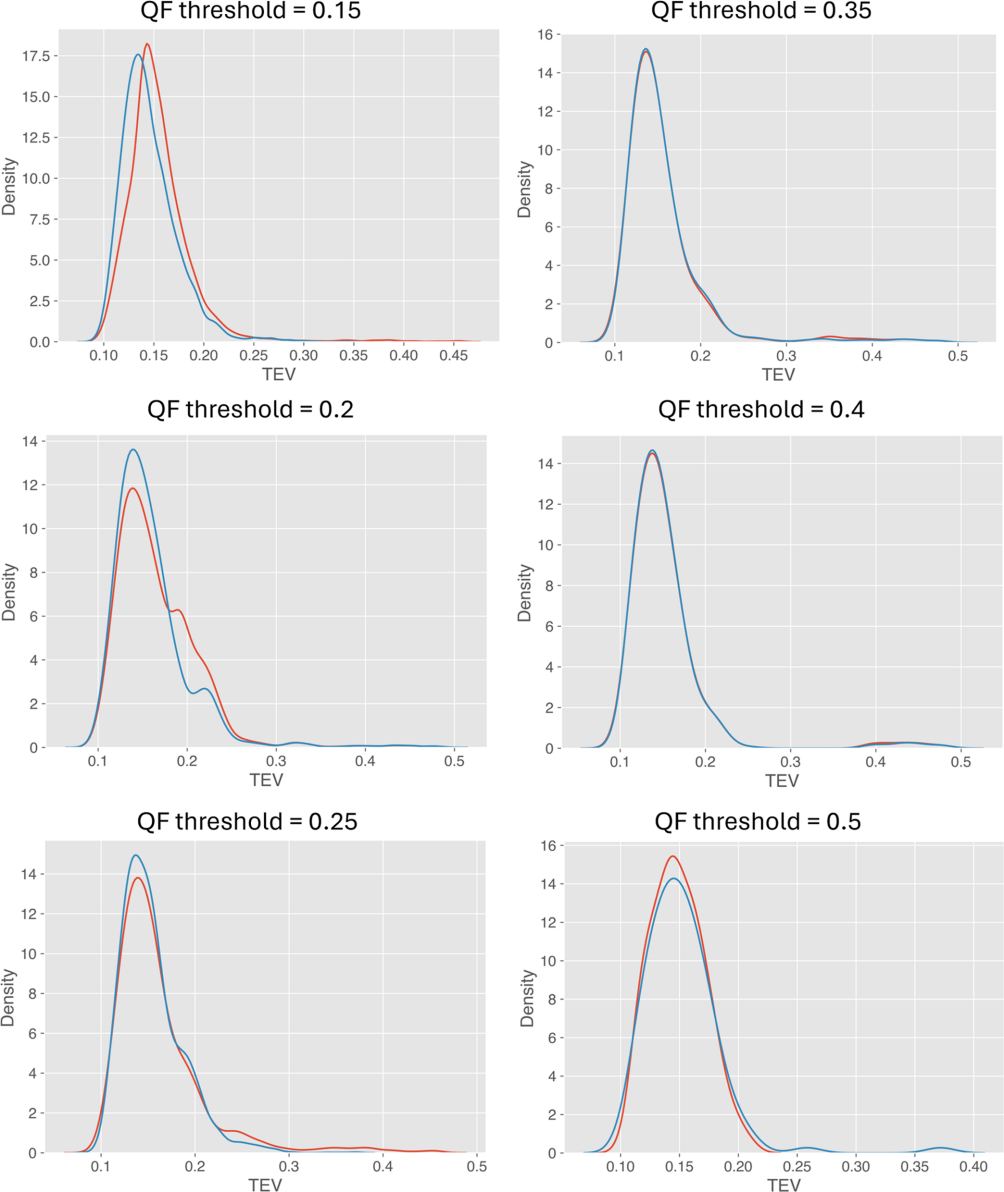
Distributions of incorrect target PSMs based on PyViscount (blue) and the synthetic (red) identification status labels at different quality filtering (QF) thresholds.

The quality filtering (QF) step executed for a range of user-selected threshold scores is capable of generating a high-quality data set necessary for validation by partition. Comparing the TEV score distributions of the PSMs labeled as incorrect by PyViscount with the synthetic reference counterparts reveals that as the QF threshold increases, the PyViscount-generated distribution becomes more similar to the reference (Figure 3). When low QF threshold values are selected, many spectra proceeding to the subset search get incorrectly identified in the full search space search. The consequence of that mislabeling is that some PSMs marked as incorrect based on the synthetic reference status are labeled as correct by PyViscount because their top-scoring peptide candidates are the same in the full and the subset search scenarios. As the QF threshold increases, the spectra that end up mislabeled get progressively sieved out, and the quality of the score distributions of incorrect PSMs generated in subset searches improves, which can be verified visually (e.g., Figure 3, panel with the QF threshold = 0.35) and by the results of the two-sided KS test (Table 1). Increasing the QF threshold, however, is beneficial only up to a certain point; when the threshold value becomes too high, fewer spectra are available for the subset search and, as a result, produce more unstable score distributions. The occurrence of this limitation is confirmed by the scenario with the QF threshold = 0.5, as shown in the density plot (Figure 3) and the corresponding value of the KS test statistic, which is higher than the values obtained for the variants with QF thresholds equal to 0.4 or 0.35 (Table 1).

**Table 1:** KS Test Results for the Quality Filtering Step

	TEV score value as a QF threshold					
KS test (two-tailed)	0.15	0.2	0.25	0.35	0.4	0.5
KS statistic	0.181	0.102	0.0413	0.0101	0.00389	0.0248
KS *p*-value	8.52*e*-107	1.63*e*-22	0.00277	0.999	1.0	1.0

### Validation of Existing FDR Estimation Methods

#### Target-Decoy Competition

**Figure 4:**
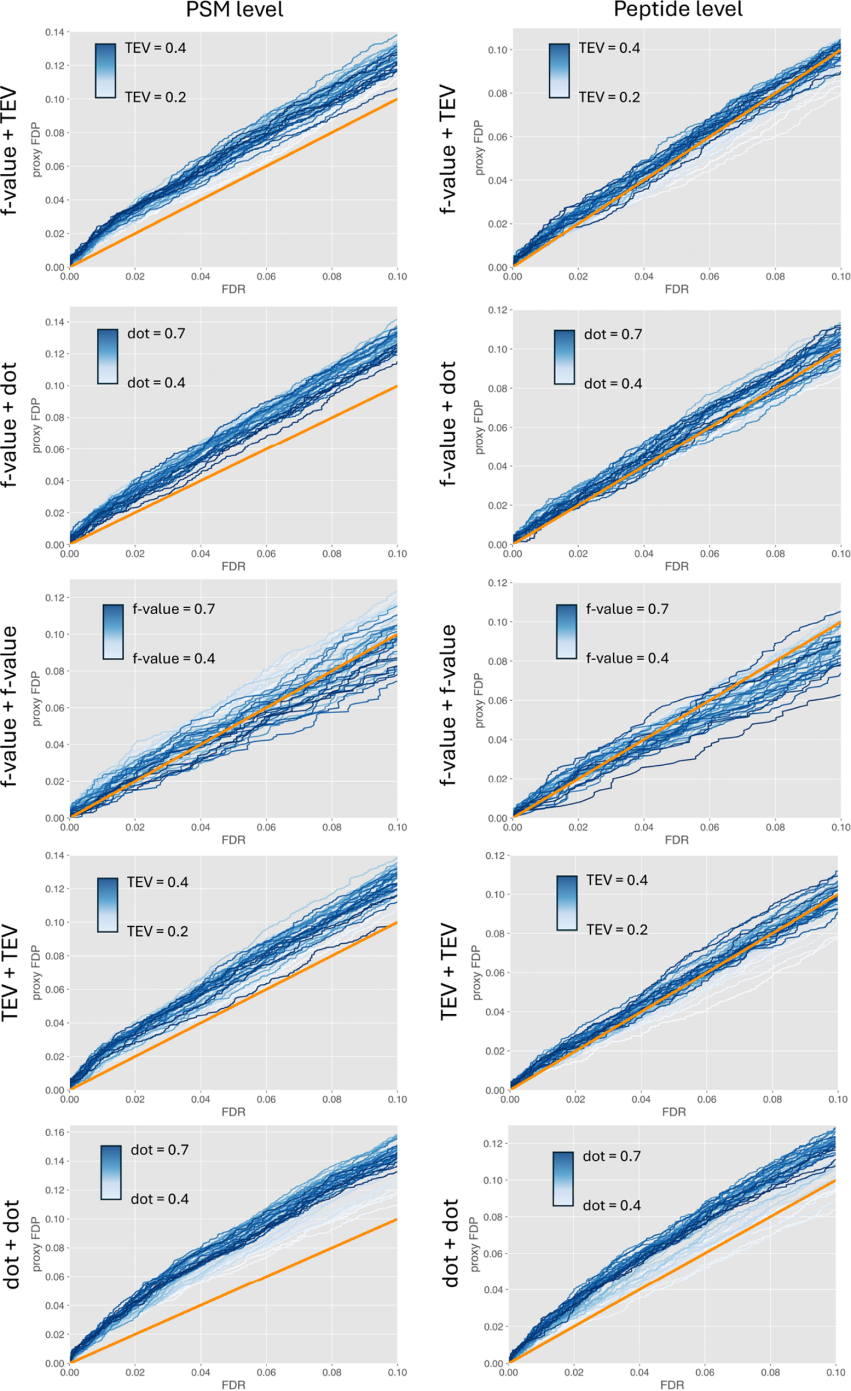
Validation of TDC-based FDR estimation at PSM and peptide levels executed on SpectraST search results using PyViscount in a postsearch partition mode, represented in the form of proxy FDP vs FDR plots (with orange *x* = *y* lines for reference). The plots (from top to bottom) correspond to scenarios where the following pairs of scores were used in the QF step and FDR estimation, respectively: *f*-value + TEV, *f*-value + dot product, *f*-value + *f*-value, TEV + TEV, dot product + dot product.

Validation by partition associated with most evaluated score combinations indicates that TDC using SpectraST decoys results in FDR underestimation (Figure 4). This observation stays consistent with past observations that the shuffle-and-reposition method^31^ produces imperfect decoy spectra, whose properties do not exactly resemble those of the target counterparts. However, the liberal nature of TDC-based FDR estimates cannot be attributed to the characteristics of the decoys used alone. The contour plot results indicate that the selection of the QF and FDR scores matters, too. Using different FDR scores impacts the performance of TDC-based FDR estimation directly, as it affects the extent of discrimination between correct and incorrect spectral matches. A different QF score does not directly influence the FDR estimation procedure, but it affects the quality of the proxy FDP—if the QF score does not have a high discriminant power, it cannot separate correct from incorrect matches well at the QF stage and may result in introducing an unknown fraction of mislabeled matches in the calculate of FDP. This is exemplified by the scenarios where SpectraST *f*-value (as defined by Lam et al.^28^ which includes dot product and dot bias terms) is the FDR score and dot product, *f*-value, and TEV scores are used for the QF step. While TEV and dot product as QF scores provide similar validation results (e.g., the observed FDP value at 10% FDR threshold is around 12% for the higher score values), *f*-value as QF score provides results suggesting that TDC-based FDR estimation for SpectraST at PSM level is closer to the expected values, but at the same time tends to be associated with larger variance across the evaluated QF score range of values. This observation may indicate that composite scores like the *f*-value based on some rather arbitrary heuristics (in this case the weights of dot product and dot bias components in the *f*-value formula) are not the best choice for the QF step in validation by partition.

Using dot product as both FDR and QF scores also seems to be a suboptimal setting. Validation based on this score combination suggests that TDC in SpectraST is even more liberal than in the other evaluated cases, including the scenario with the dot product as the QF score but a different FDR score (*f*-value). It means that the observed underestimation of FDR could be caused by decoy candidates generally obtaining lower dot product scores than target candidates mapped to incorrect spectral matches. This phenomenon could be attributed to either the problematic nature of the decoy generation method or the limited compliance of the dot product scoring function with the assumptions behind TDC. The trends exhibited by the validation protocols using different QF and FDR score combinations are also reflected in the results of FDR estimation at the peptide level. However, the magnitude of FDR underestimation seems to be smaller, which is consistent with the previous studies on the characteristics of TDC, which demonstrate that TDC has a tendency for liberal FDR estimation at the PSM level but, according to theory, can control FDR at the peptide level.^42^

#### Storey’s Method with Decoy-Based *p*-Values

**Figure 5:**
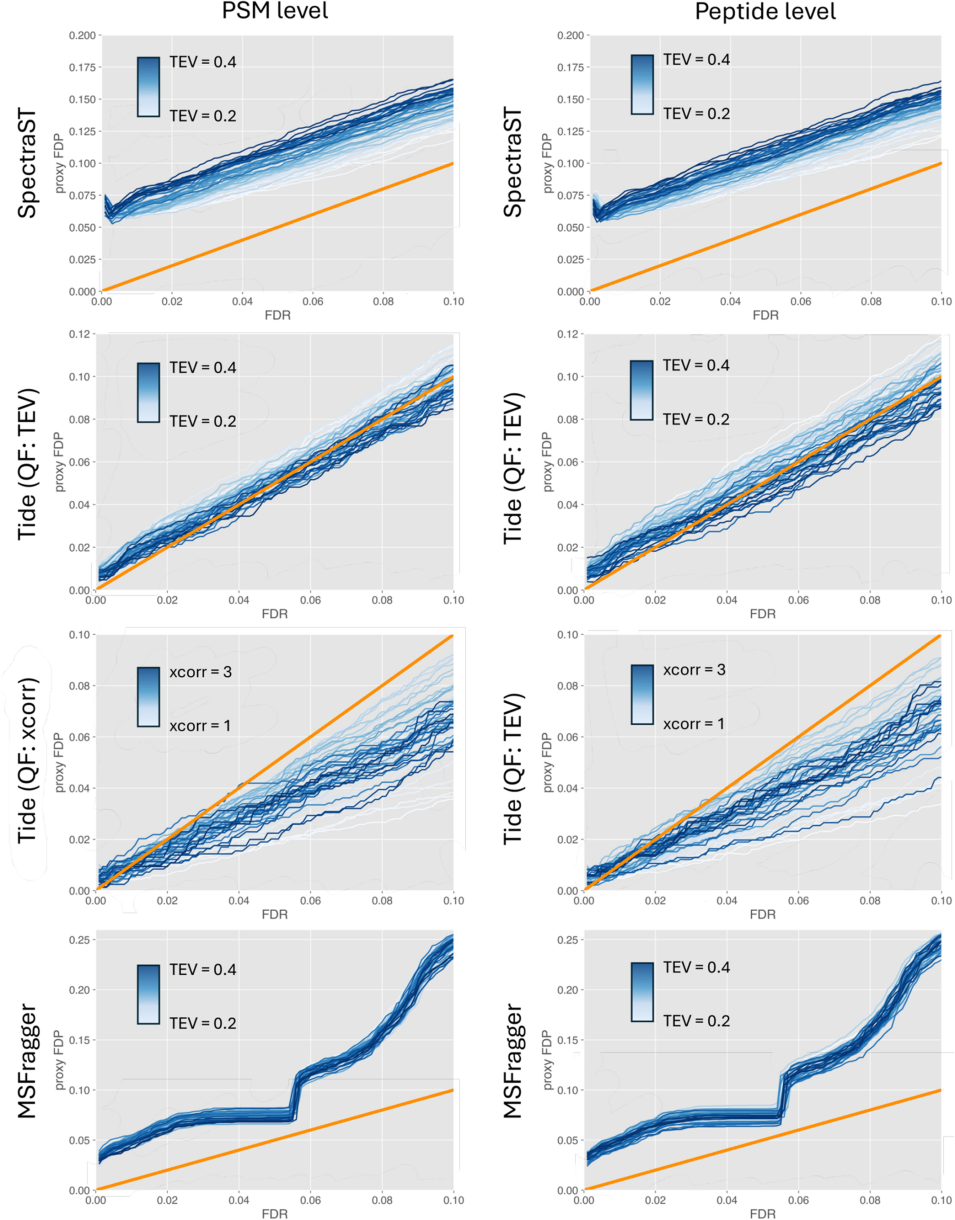
Validation of Storey’s FDR estimation method at PSM and peptide levels executed on SpectraST, Tide (two cases with TEV and *x*corr scores used in the QF step), and MSFragger search results using PyViscount in a postsearch partition mode, represented in the form of proxy FDP vs FDR plots (with orange *x* = *y* lines for reference).

FDR estimates produced by Storey’s procedure utilizing *p*-values derived from decoy PSMs score distribution differ substantially depending on which search engine is used to produce the analyzed PSMs (Figure 5). In all evaluated scenarios, there is virtually no difference between the overall trend and the bias exhibited for different FDR threshold values on PSM and peptide levels. It is not the default behavior of an FDR estimation method. On the PSM level, an FDR estimation procedure needs to deal with the fact that multiple PSMs can originate from the same peptide, and thus their scores are often not independent from each other. The scale of this problem is substantially decreased on the peptide level, where only one PSM per putative peptide is considered in the estimation. Therefore, it is not uncommon for FDR estimation procedures to exhibit different trends at the PSM and peptide levels. As a result, the community seems to discourage the use of PSM-level FDR estimation in favor of the several variants of peptide-level alternatives.^18,43^ However, the results presented in Figure 5 indicate that Storey’s method is largely unaffected by the dependency of scores (*p*-values) used in FDR estimation.

The performance of each investigated search engine for Storey’s method is similar to what is observed in the already discussed scenario of TDC-based FDR estimation. SpectraST and MSFragger do not provide accurate FDR estimates again, and they tend to return substantially underestimated values. However, the characteristics of the trend in the FDR estimated using Storey’s method provide additional insights into what might cause the inaccurate FDR estimation for these two search engines. For SpectraST, the relationship between FDR estimates and the corresponding FDP values is roughly linear and parallel to the expected trend (*x* = *y* line), indicating an impact of a simpler effect concerning all spectral matches. For MSFragger, the trend appears nonlinear and rather irregular, suggesting that the associated effect is more complex and might not affect all PSMs equally. Based on these observations, it could be speculated that SpectraST’s problem lies in the quality of decoy spectra which tend to obtain scores that are consistently lower than expected. The issue with MSFragger is likely much deeper - it seems to be associated with the scoring process itself and could be caused by heuristics applied to the scoring functions or by extra pre- or postprocessing steps aimed at improving the discriminating performance of the scores (such solutions are implemented in MSFragger as evidenced by the availability of the optional score “boosting” feature^40^).

The performance of Storey’s method also depends on the underlying score used in the calculation of decoy-based *p*-values, as demonstrated by the results obtained for Tide when TEV and refactored xcorr scores were used (Figure 5). The FDR estimates obtained for *p*-values derived from the decoy distribution of refactored xcorr score tend to be more conservative than the TEV counterparts. This discrepancy could be explained by the fact that the refactored xcorr score is not calibrated, and thus cannot be reliably used to compare PSMs originating from different spectra.^44^ While the scenario where the TEV score is used in the calculation of *p*-values exhibits less conservative FDR estimates, it is consistent with the validation results obtained for TDC and thus seems to reflect the actual performance of the evaluated FDR estimation method.

#### Decoy-Free Methods

**Figure 6:**
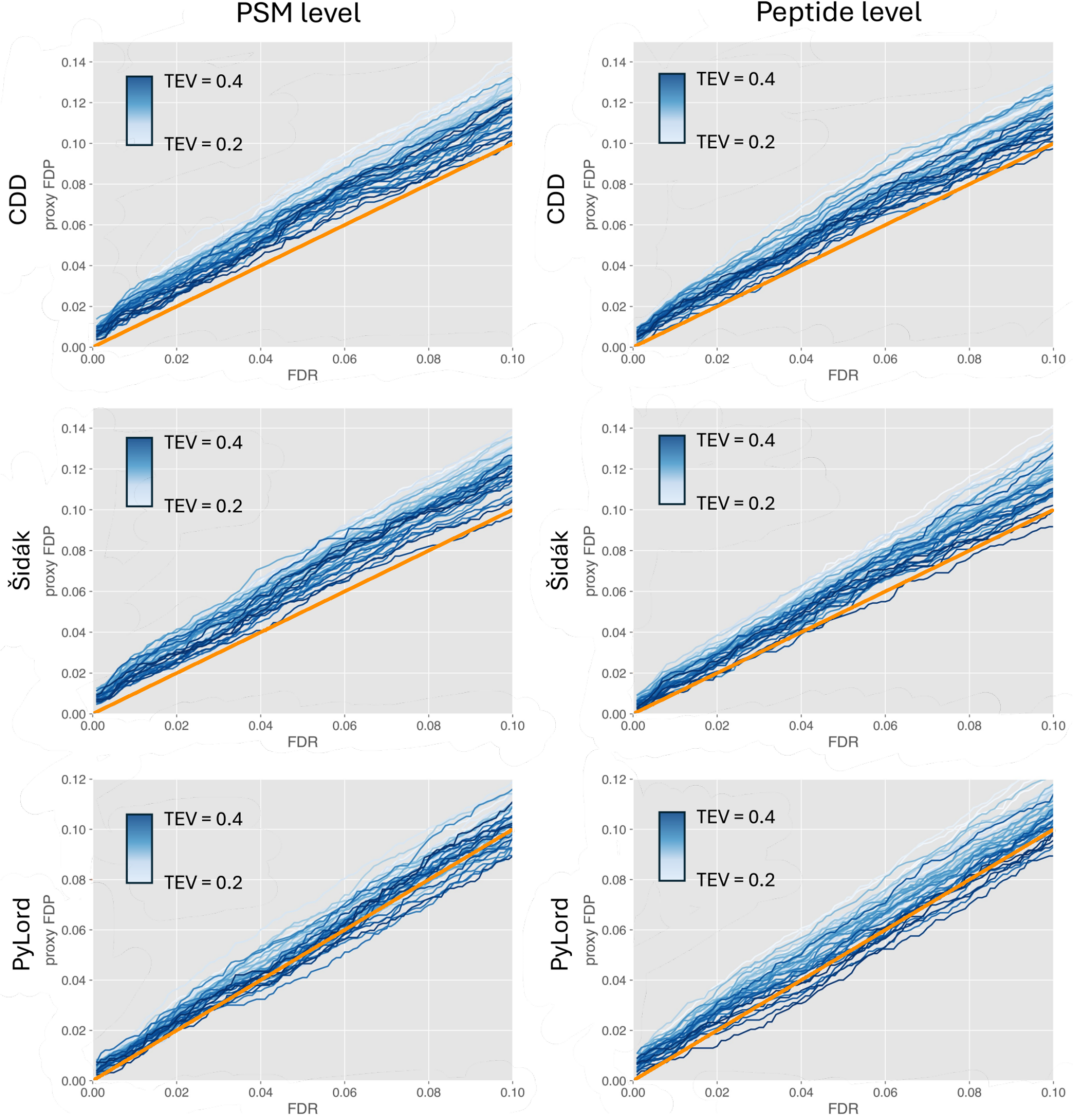
Validation of Storey’s FDR estimation method executed on CDD, PyLord, and Šidák-corrected spectrum-level *p*-values obtained for Tide search results using PyViscount in a postsearch partition mode (with orange *x* = *y* lines for reference).

The results shown in Figure 6 demonstrate that Storey’s procedure using CDD and Šidák-corrected spectrum-level *p*-values slightly underestimates FDR on both PSM and peptide levels across the whole evaluated FDR threshold range. The rather consistent liberal bias is likely a consequence of how CDDs are constructed. Millions of decoy PSMs generated using previously acquired experimental data are supposed to capture the “global” behavior of incorrect matches which substantially decreased variance. However, that advantage comes at the cost of a likely increase in bias when the “global” CDD is used for estimating FDR on the “local” level of an individual data set. This intuition seems to be confirmed by validation results produced by PyViscount. Another factor that could contribute to the slightly liberal bias exhibited by the variants using the CDD but also the Šidák-corrected spectrum-level and PyLord-based *p*-values is the potentially inaccurate π_0_ estimate used in the FDR formula in Storey’s procedure. Conventionally, the π_0_ in the proteomics context is defined as the fraction of incorrect PSMs among all analyzed PSMs. However, in virtually all implementations it considers only incorrect matches due to foreign spectra, i.e., spectra that originate from entities absent from the selected search space; typically, such spectra are associated with contaminants or peptides with modifications not considered during the search. Most methods of estimating π_0_ ignore the incorrect matches due to native spectra whose correct candidates are in the search space but for whatever reason got outscored by other target candidates.^45^ Therefore, the π_0_ commonly used in Storey’s procedure or separate target-decoy search protocols is, to some extent, underestimated, which eventually entails liberal FDR estimates.

The upward bias seems to be minimally smaller for the variant using PyLord-based *p*-values, which provides the most accurate FDR estimates at both PSM and peptide levels among the investigated methods (Figure 6). This improvement in accuracy could be attributed to the fact that PyLord takes advantage of multiple lower-order TEV score distributions to help estimate the score distributions of incorrect top-scoring target PSMs for each charge state separately.^37^

### Comparison with Other Validation Protocols

#### Entrapment Database Method

**Figure 7:**
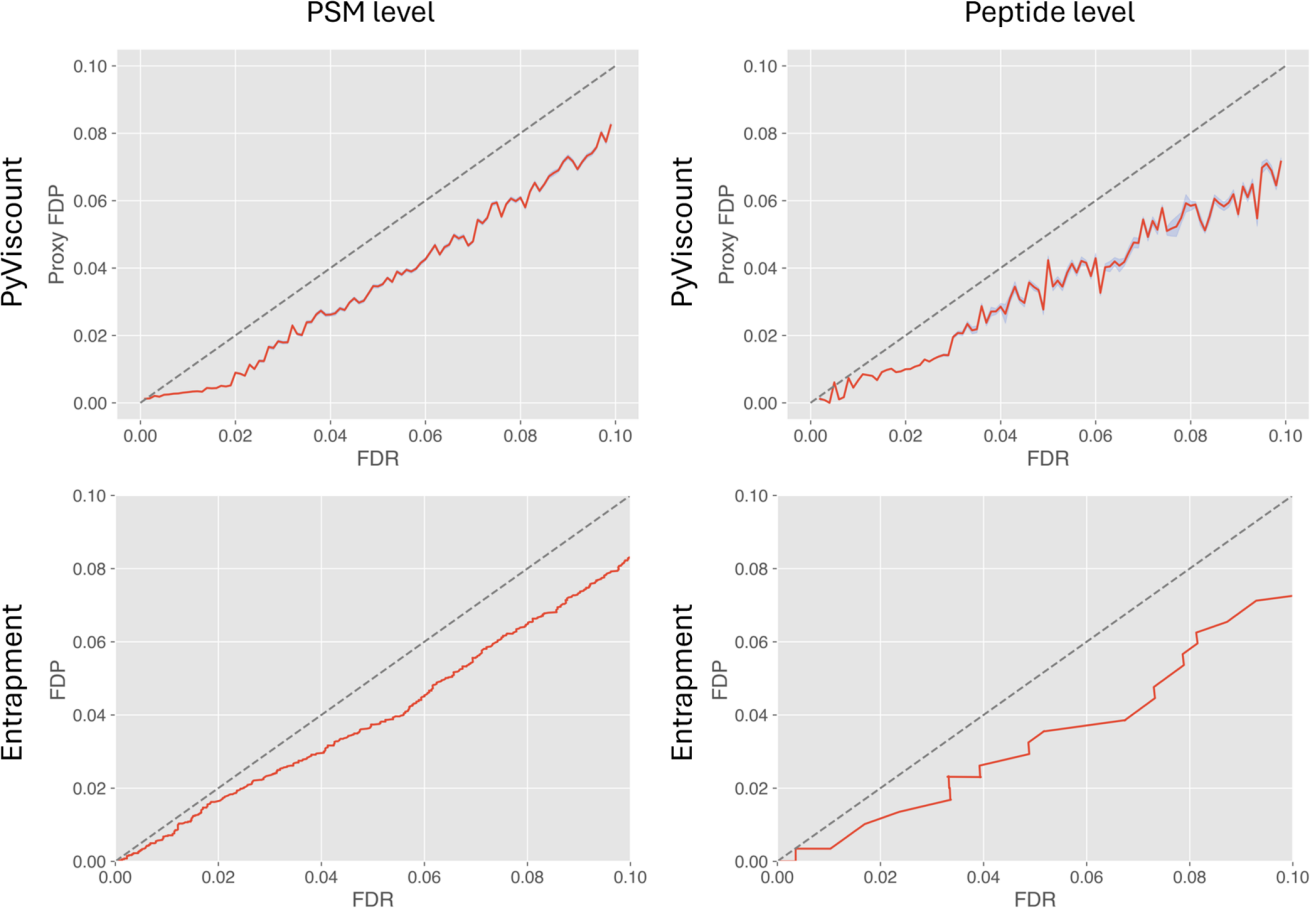
Results of validating TDC-based FDR estimation at PSM and peptide levels obtained from PyViscount and entrapment protocols (with gray *x* = y lines for reference). The PyViscount plots represent the results obtained using optimal QF thresholds and 5479 and 5569 spectra on PSM and peptide levels, respectively. The entrapment protocol utilized 34,457 spectra on both PSM and peptide levels.

Generally, the validation results provided by PyViscount are consistent with those generated by the protocol based on an entrapment database (Figure 7). On the PSM level, the trend in FDR estimation is very similar between these validation methods across most of the evaluated FDR range. However, a small difference can be observed at the low FDR threshold region (0.1–2%) and it could be explained by several factors. First, the right tail of the score distribution of incorrect PSMs is usually sparse, i.e., that region has fewer data points, which also tend to be scattered more than the points in other regions characterized by larger probability values, e.g., the mode of the distribution. In the context of entrapment database methods, the scale and direction of that effect may also differ depending on the type of entrapment database used.^6^ Second, the actual data set used to generate the proxy FDP vs FDR plot by PyViscount is a subset of the original set of spectra employed in the validation using an entrapment database, and due to its smaller size and random nature of partition can produce slightly different validation results for each subset belonging to the partition. Therefore, it is expected that the validation results obtained using these two approaches will differ to some small extent simply due to the randomness associated with the partition and scoring steps.

Although it is difficult to say with certainty whether there are some other sources of differences between the partition- and entrapment-based validation results other than randomness, it could be argued that the slightly more liberal trend of the entrapment FDR estimates at the low FDR threshold region stems, to some extent, from the large evolutionary distance between the entrapment and the sample organisms. While the small overlap between the human and *Archaea* species ensures that matches to entrapment sequences are incorrect, which is one of the requirements for any entrapment protocol, it is also associated with lower homology between sequences from these two proteomes. That difference may cause the top-scoring entrapment matches to model the behavior of the top-scoring incorrect matches inaccurately because low-homology entrapment candidates may often be incapable of reaching similarity scores as high as those of the high-homology sample candidates. Therefore, the FDP values determined in an entrapment database framework may sometimes be slightly too high at the low FDR threshold range. Nevertheless, the magnitude of this effect appears rather negligible and should not dramatically distort the respective validation results in practice.

The relationship between (p)FDP and FDR estimates at the peptide level is slightly more jagged than at the PSM level for both investigated methods (Figure 7, right column). This effect is probably caused by the smaller number of data points available for calculation since the number of peptides is necessarily smaller than the number of corresponding PSMs. Nevertheless, the trends exhibited by both methods are very similar and do not differ from those obtained for the PSM-level analysis. Overall, the results of validating TDC-based FDR estimates using the fully labeled entrapment database protocol and validation by partition are consistent with each other across most of the evaluated FDR threshold values. The similarity of the results obtained from the two validation protocols with vastly different approaches to determining FDP suggests that both methods, in this particular context, could be considered reasonably reliable because it is quite unlikely to obtain results of such high similarity from two incorrect protocols.

#### Intersection + Precursor Mass Shift

**Figure 8:**
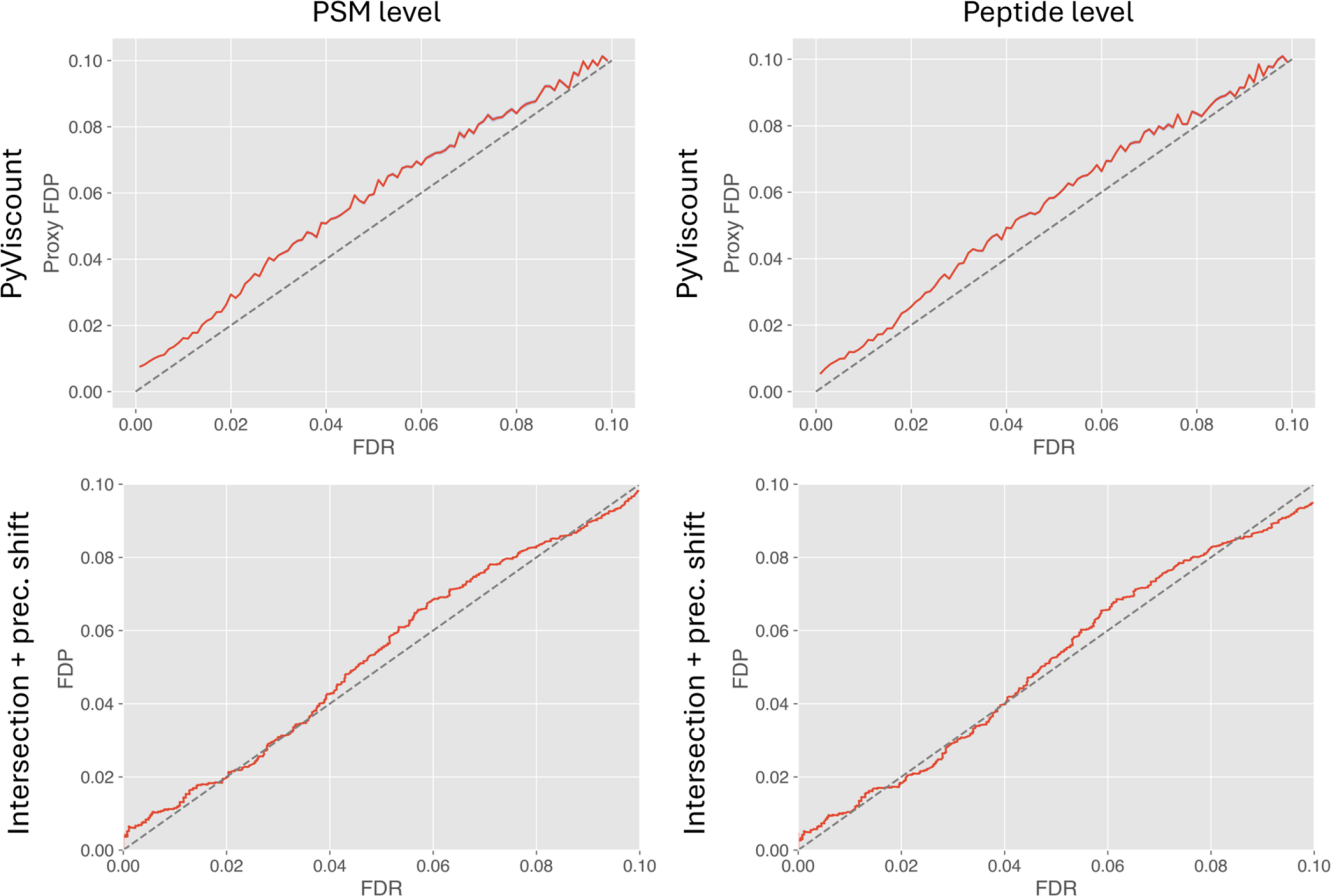
Results of validating TDC-based FDR estimation at PSM and peptide levels obtained from PyViscount and intersection + precursor mass shift protocols (with gray *x* = *y* lines for reference). The PyViscount plots represent the results obtained using optimal QF thresholds and 4346 and 3238 spectra on PSM and peptide levels, respectively. The intersection + precursor mass shift protocol utilized 16,505 spectra on both PSM and peptide levels.

The assessment of TDC-based FDR estimation by the validation by partition and the intersection-precursor mass shift protocols suggests that the validation trends exhibited by both methods are similar (Figure 8), although several minor differences can be observed. Generally, the FDR estimates on PSM and peptide levels do not differ too much from each other in the case of both validation protocols, however, the peptide-level estimation appears relatively more conservative than the PSM-level variant. Interestingly, in the case of intersection-precursor mass shift protocol, the FDR estimates are very close to the expected values in the low FDR threshold range (0.1–4%), while for larger thresholds, the tendency to underestimate is observed. Validation by PyViscount, on the other hand, suggests the liberal FDR estimation trend with a slightly larger magnitude of the upward bias occurs across all evaluated FDR thresholds.

The difference between the results provided by these two validation methods could be explained by the fact that the intersection-precursor mass shift protocol utilizes a data set of a dramatically different nature, i.e., a set of natural spectra that get matched to the same peptide candidate by multiple search engines (positive samples) and the spectra with artificially shifted precursor mass, which are bound to produce incorrect PSMs (negative samples). PyViscount, on the other hand, relies on subset-searching only the high-quality portion of that data set (generated in the quality filtering step), which happens to be the set of the natural, unmanipulated spectra. Therefore, it could be argued that intersection-precursor mass shift protocol provides more “perfect” validation results due to the artificially well-behaved incorrect PSMs subjected to the analysis, while PyViscount deals with the more realistic, and thus less well-behaved case of the subset of real spectra. It is difficult to judge which validation protocol provides a more reliable assessment of the accuracy of TDC-based FDR estimation for the analyzed data set simply because the absolute truth about the actual identification status of each evaluated PSM is unknown. Nevertheless, it seems reasonable to admit that both protocols provide results that suggest the existence of the same trend, i.e., that in this particular scenario, FDR estimates produced by TDC have the tendency to be slightly underestimated. Thus, as in the case of comparison with the entrapment databased protocol, the similarity between the results of the validation by partition and the intersection-precursor mass shift method seems to suggest that, in this particular context, both methods could be treated as reasonably reliable because obtaining results of such high similarity is more likely for two correct than for two incorrect validation protocols.

#### Verification of the Synthetic Origin

**Figure 9:**
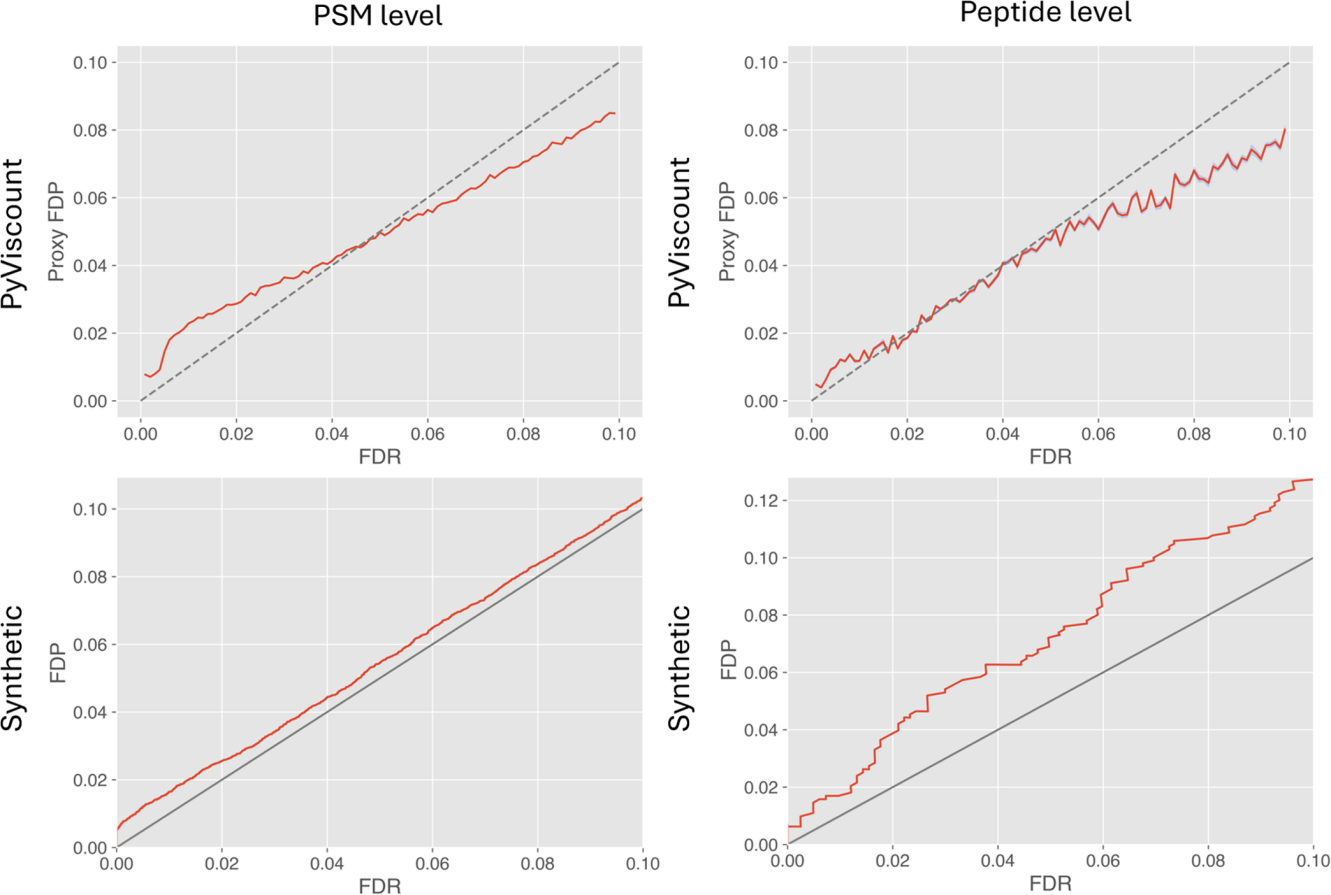
Results of validating TDC-based FDR estimation at PSM and peptide levels obtained using PyViscount and verification of synthetic origin protocols (with gray *x* = *y* lines for reference). The PyViscount plots represent the results obtained using optimal QF thresholds and 21,632 and 24,155 spectra on PSM and peptide level, respectively. The verification of synthetic origin protocol utilized 54,297 spectra on both PSM and peptide levels.

Comparison of validation by partition with the protocol based on verification of synthetic origin reveals that the two methods provide strikingly different assessments of TDC-based FDR estimation on both PSM and peptide levels. At the PSM level, the latter indicates that the FDR is consistently underestimated, with the bias slightly decreasing as the FDR threshold increases (Figure 9, left column). The peptide-level analysis, however, displays a substantially different relationship between FDP and the FDR estimates, suggesting a stronger liberal trend in the TDC-based FDR estimation process, with the bias sharply increasing for larger FDR values (Figure 9, right column). It is a somewhat surprising finding because while TDC does not guarantee FDR control at the PSM level (and instead often leads to underestimation of FDR), it does, at least according to theory, control FDR at the peptide level.^42^ The trend observed in the validation by verification of synthetic status executed on the spectral data set of synthetic peptides seems to exhibit the opposite tendency - FDR estimated at the peptide level is more liberal than at the PSM level.

Validation by partition provides results that appear more consistent with each other but hint at the existence of potential problems with the analyzed data set. At the PSM level, the validation protocol suggests underestimation of FDR in the lower FDR values region (0.1–5%) and overestimation in the upper region (Figure 9). The FDR estimates obtained from the peptide-level analysis are also split into two sections below and above the 5% FDR value. Below that threshold, the estimated FDR values are very close to the proxy FDP values, while above the 5% FDR threshold, they are conservative to a larger extent than the corresponding values on the PSM level. The resemblance of FDR estimation trends on the PSM and peptide levels in the results provided by the validation by partition seems consistent with the theoretical properties of TDC and makes this validation protocol appear a bit more sensible than the verification of the synthetic origin.

The strong discrepancy between the results returned by the two validation protocols compared in this section could be partially attributed to the fact that validation by partition utilizes only a subset of the synthetic spectral data set used in its entirety in the validation by verification of synthetic origin. However, the existence of the peculiar 5% FDR “pivot point” in the plot produced by PyViscount could suggest that there is some problem with the analyzed data set itself, perhaps related to the heterogeneity of the produced PSMs influenced by the imperfections and the design of the experimental protocol^11^ applied in synthesizing the peptides used to generate the analyzed mass spectra. It is also possible that uninterpretable spectra from the synthetic data set originate, at least to some extent, from mis-synthesized sequences with swapped or missing amino acids, which could be highly homologous with the target sequences, leading to target bias violating the equal chance assumption of TDC.^38^ Regardless of the identity of the factors leading to such different validation results provided by the two methods tested here, it is not unreasonable to suspect that utilizing synthetic data sets, especially those obtained from low-complexity peptide mixtures, may lead to peculiar and rather unrealistic scenarios that give rise to questionable results when used in the validation of an FDR estimation method.

## Conclusions

Validation by random search space partition enables flexible assessment of various FDR estimation methods. The framework is compatible with different search spaces (sequence databases, spectral libraries) and generic experimental spectral data sets, provided they have a sufficiently large number of identifiable spectra. It allows the users to gain deeper validation insights by providing two forms of reporting, contour and optimized single-line plots, which can be easily specified using an option in the configuration file. Analysis conducted in this study confirms that validation by search space partition provides results consistent with the knowledge we already have about the popular FDR estimation methods and stays in agreement with some existing validation procedures. Thus, the proposed protocol could be an attractive alternative available to proteomics practitioners who would like to gain more insights into the performance of the FDR estimation methods across a wide range of realistic testing scenarios.

While the validation by search space partition is free from shortcomings typical for the existing validation protocols involving query or search space manipulation, it still possesses some limitations. It may not provide reliable results for extremely small search spaces or query spectra sets; however, this constraint may negatively affect the performance of any validation protocol currently available in proteomics. An issue that stems from the method’s reliance on the binary nature of the identification status (correct/incorrect) is the lack of compatibility with chimeric spectra,^46^ which may have more than one correct candidate matched. An optimal treatment of such cases in a validation process remains an open question. A somewhat related problem that limits the application scope of validation by search space partition is the fact that some search spaces may not be compatible with this validation paradigm. The most obvious example of such search space is a spectral archive^47^ characterized by the candidate multiplicity, i.e., the search space has multiple instances of candidates mapped to the same peptide sequence. While collapsing the candidate list for each query spectrum into a set of unique peptide candidates could make the spectral archive search results compatible with the proposed validation framework, the optimal way of achieving that while preserving the advantages of using the candidate multiplicity is up for debate.

Overall, we believe that the validation by partition, as implemented in PyViscount, constitutes a much-needed contribution to the field as it draws attention to the underdeveloped and largely unstandardized state of validating FDR estimation methods in proteomics. By focusing on minimal interference with the natural characteristics of the analyzed queries and the search space used, PyViscount makes it feasible to obtain reliable validation results without resorting to synthetic and more unrealistic scenarios.

## Data Availability

The source code of PyViscount is available at https://github.com/dommad/pyviscount.

## Supporting Information Available

Score adjustment in validation by search space partition (Note S1); reporting PyViscount results: contour plot (Note S2); example of a PyViscount contour plot (Figure S1); reporting PyViscount results: optimized single-line plot (Note S3); optimization of the quality score threshold (Figure S2); example of a PyViscount optimized single-line plot (Figure S3) (PDF)
