# Supplementary material for: PyViscount: Validating False Discovery Rate Estimation Methods via Random Search Space Partition
Source: J Proteome Res. 2025 Feb 5;24(3):1118–34. doi: 10.1021/acs.jproteome.4c00743 (PMC11894659; doi:10.1021/acs.jproteome.4c00743)

Supporting Information for the article: ”PyViscount: Validating False Discovery Rate Estimation Methods Via Random Search Space Partition”

Dominik Madej, Henry Lam

Department of Chemical and Biological Engineering, the Hong Kong University of Science and Technology, Hong Kong, China

Content

Supplementary Note S1: Score adjustment in validation by search space partition

Supplementary Note S2: Reporting results: contour plot

Supplementary Figure S1: Example of a PyViscount contour plot

Supplementary Note S3: Reporting results: optimized single-line plot

Supplementary Figure S2: Optimization of the quality score threshold

Supplementary Figure S3: Example of a PyViscount optimized single-line plot

Supplementary Note S1 Score adjustment in validation by search space partition

Validation in the post-search mode does not involve an explicit partition of the search space prior to the search, and instead relies on the ”simulation” partition subset search executed on the existing results of searching the full search space. While both pre- and post-search partition modes can generate equivalent validation results, the post-search variant may require additional modifications, depending on the scoring functions used and the false discovery rate (FDR) estimation method evaluated.

The first special case occurs when the FDR estimation method to be tested relies on competition between targets and external negative samples in the search space. A typical example of such a case is validating target-decoy competition (TDC). To ensure correctness of such validation, the post-search mode requires additional adjustment of the validation score and special treatment of decoys during assignment of identification status labels and calculation of proxy false discovery proportion (pFDP) and FDR estimation.

The reason behind the necessity of validation score adjustment is that, for the sake of conceptual simplicity, the decoy section of the search space is not partitioned during the post-search mode. In other words, the ”simulated” partition subset search involves searching the target candidates belonging to selected search space subset and the decoy candidates belonging to the decoy equivalent of full search space. Consequently, the effective sizes of the decoy and target databases used at the stage of ”simulated” subset search are different and depend on the number of the equal-sized subsets comprising the partition P_N. That, in turn, affects the values of similarity scores which are designed to adjust for the search space size. One of such scores is the log-transformed e-value (TEV), defined in the main manuscript. In the setting of sequence target-decoy database search, with the ratio of sizes of decoy to target databases R_{TD} and the partition consisting of N equal-sized subsets, the

search results obtained after the ”simulated” partition subset search must have their TEV scores adjusted according to the formula:

$$\text{TEV}_{sub} = \text{TEV}_{full} - \beta \times \ln\left(1 - \frac{1}{N \times (1 + R_{TD})}\right) \tag{1}$$

where TEV_{sub} is the adjusted TEV score, TEV_{full} is the TEV score before adjustment, and β is the user-defined parameter used in the calculation of the original TEV scores.

In the case of target-decoy competition validated via the post-search mode, the process of identifying the top-scoring candidate C_h for each spectrum $S \in Q_f$ (where Q_f is the set of spectra that passed the quality filtering step) in the ”simulated” subset search must be amended to accommodate the possibility of a decoy hit as well. If it happens that for spectrum $S \in Q_f$, there is a decoy candidate outscoring the top target candidate belonging to the selected subset of the search space partition, the decoy candidate is recorded as C_h and is not assigned any identification status label since it is going to be used only in FDR estimation, not in pFDP calculation.

Practically, in the post-search mode, the FDR estimation via target-decoy competition using decoy counting can be executed according to the following formulas:

$$\widehat{\text{FDR}} = \frac{N_D(x) + 1}{N_S N_T(x)} \tag{2}$$

where N_s is the number of subsets in search space partition. The rationale behind using the corrective factor N_s is that an incorrectly identified spectrum is equally likely to be mapped to a target or a decoy candidate. Consequently, the ratio of decoy to target hits above the selected validation score threshold should equal the ratio of decoy to target database sizes. Since in the proposed post-search mode a full decoy search space and one subset from equally partitioned target search space are used, it corresponds to N_S.

The remaining types of FDR estimation methods (decoy-free and those based on separate target-decoy searching) also require adjustment of scores that correct for the size of

the search space used. They do not involve any competition, therefore the adjustment only needs to account for the numbers of sets in the partition only. The necessary correction can be demonstrated on one of such scores, the already mentioned the TEV score. In the case of decoy-free methods executed on target-only search results, the situation is straightforward. Searching a partition subset means that the original TEV score needs to be adjusted by a factor corresponding to the number of equal-sized subsets comprising the partition:

$$\mathrm{TEV}_{sub} = \mathrm{TEV}_{full} - \beta \times \ln\left(1 - \frac{1}{N_S}\right) \tag{3}$$

The same formula be be applied to TEV scores used in validating FDR estimation using separate target-decoy search results. In this case, the TEV scores for targets and decoys are calculated in two separate searches. And since the post-search mode does not involve partitioning the decoy search space, the TEV score adjustment needs to be applied only to the target TEV scores corresponding to the matches produced in the ”simulated” partition subset search.

Supplementary Note S2 Reporting results: contour plot

The contour plot representation of the validation results is particularly beneficial as it is capable of providing deeper insights into the trends exhibited by the tested FDR estimation method. Also, it is an intuitive choice when the results comprise data obtained from validation executed for a range of quality filtering threshold values. The number of lines in the contour plot depends on the number of threshold values specified by the user in the validation settings. An example of a contour plot produced for validation by partition is shown in Supplementary Figure S1.

Supplementary Figure S1: Example of a contour plot with the lower and upper bounds of quality filtering (QF) score thresholds shown.

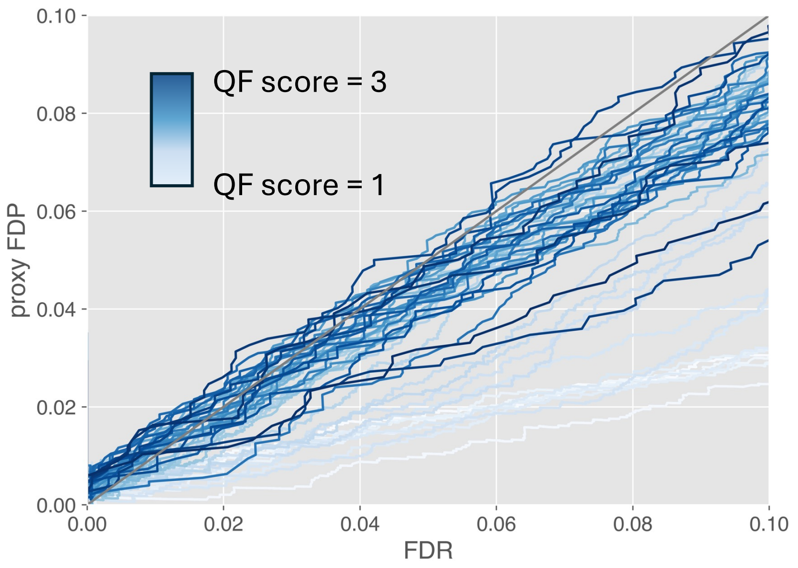

Supplementary Note S3 Reporting results: optimized single-line plot

The contour plot may not be the optimal choice when the user is not interested in the comprehensive analysis of the trends exhibited by the FDR estimation method, but instead requires the accurate and reliable results obtained for the identification status labels of sufficiently high quality. In that case, the contour plot needs to be transformed by discarding the lines corresponding to the low-confidence and high-variability scenarios. In other words, determining the optimal quality filtering threshold range requires identifying the thresholds for which high-confidence, low-variability validation results are obtained.

Practically, that task can be executed as follows. First, The FDP vs. FDR results are discretized by allocation to N_b bins in the evaluated FDR threshold range R_{FDR} (in PyVis-

count R_{FDR} by default equals to $[0.001, 0.1]$). For each FDR bin b_i, the average FDP value $\overline{\mathrm{FDP}_i}$ is calculated. Then, for each quality score threshold T_j in the threshold range R_T, the numerical approximation via backward differences of the following partial derivative is calculated:

$$f(T, \mathrm{FDP}, \mathrm{FDR}) = \frac{\partial T}{\partial \mathrm{pFDP}}|_{\mathrm{FDR}=b_i} \approx \frac{T_{j+1} - T_j}{\mathrm{pFDP}_{i+1} - \mathrm{pFDP}_i}|\mathrm{FDR} = b_i \tag{4}$$

Alternatively, the numerical approximation can be executed using forward and central differences. Next, for each quality score threshold T_j, the mean values of the absolute values of $f(T, \mathrm{FDP}, \mathrm{FDR})$ are calculated:

$$M(T_j) = \frac{1}{N} \sum_i^N |f(T, \mathrm{pFDP}, \mathrm{FDR} = b_i)| \tag{5}$$

The function $M(T_j)$ is particularly useful because it represents the magnitude of how "fast" the threshold values change with respect to the corresponding FDP values. The smaller that change, the most stable the validation which can be achieved only with high-confidence identification status labels. However, the numerical nature of the approximation often makes the resulting $M(T)$ values relatively noisy, especially when executed on small T increments. For this reason, to expose the underlying trends better, the $M(T)$ data is subjected to smoothing by applying moving average with the heuristically determined window size equal to $0.1|R_t|$. The optimal value of quality score threshold T_{opt} can be obtained by calculating the following:

$$T_{opt} = \arg\min_T M(T) \tag{6}$$

In the analysis of real data, finding T_{opt} can be facilitated by limiting the region of T considered. It can be achieved by using the fact that in a simplified real scenario, the plot of $M(T)$ is associated with two big bumps. The bump associated with lower $M(T)$ value corresponds to the scenario in which most mislabeled matches are filtered out and

the quality of validation increases rapidly. The second major bump appears when quality threshold becomes too stringent, most of the spectra are filtered out and the size of spectral dataset used for validation is very small, leading to unstable validation results. The region in between these two bumps is associated with validation on high-quality datasets and constitutes the optimal T range (summary presented in Supplementary Figure S2).

Supplementary Figure S2: Optimization of the quality score threshold range selected for reporting single-line plot of validation by search space partition.

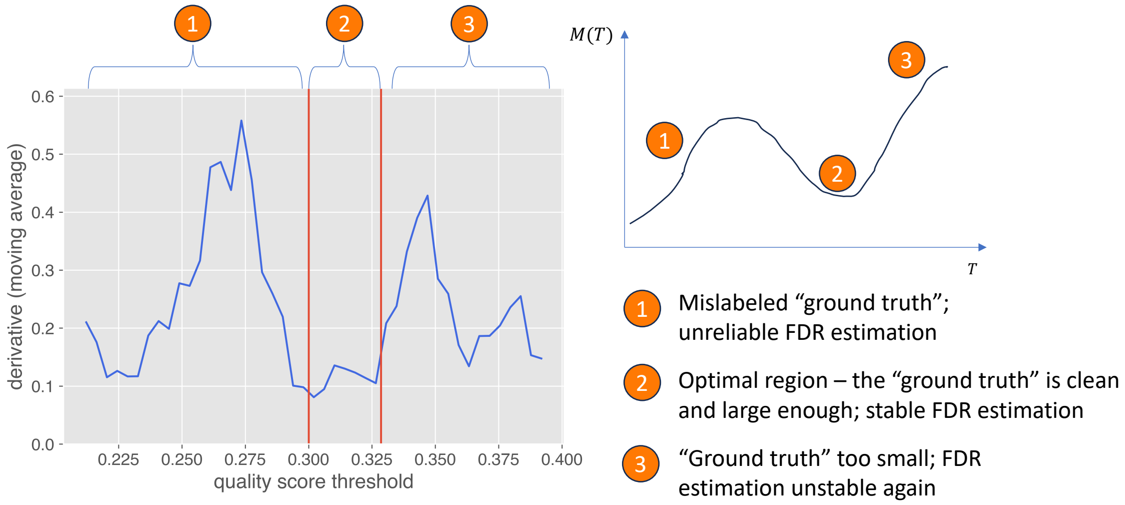

Once the location of the optimal threshold T_{opt} is determined, the validation is repeated 100 times for that threshold value. The resulting FDP values are averaged and plotted against the FDR thresholds as a single line along with the 68% pointwise confidence band. Example of such optimized single-line plot is shown in Supplementary Figure S3.

Supplementary Figure S3: Example of a single-line plot with 68% pointwise confidence band.

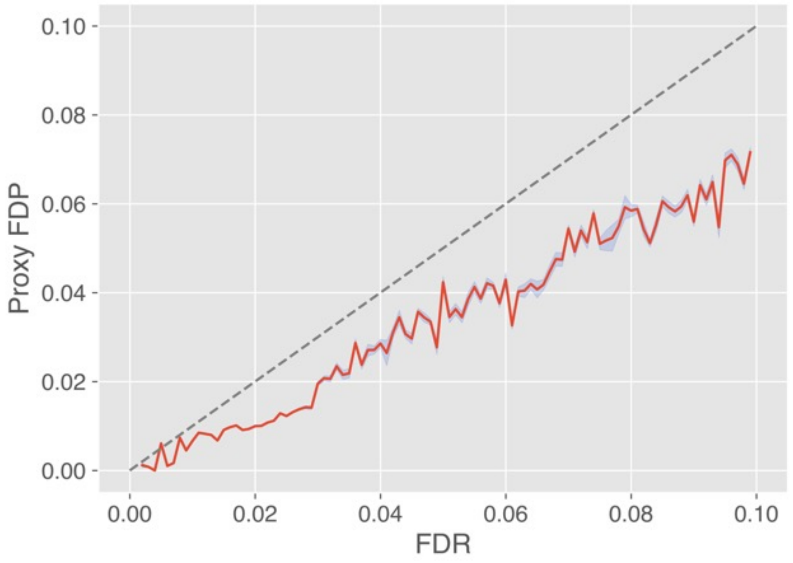

Supplement: Supplementary file 1 — pr4c00743_si_001.pdf [file pr4c00743_si_001.pdf]
